# The role of charged residues in the transmembrane helices of monocarboxylate transporter 1 and its ancillary protein basigin in determining plasma membrane expression and catalytic activity

**DOI:** 10.1080/09687860600841967

**Published:** 2006-12-13

**Authors:** Christine Manoharan, Marieangela C. Wilson, Richard B. Sessions, Andrew P. Halestrap

**Affiliations:** Department of Biochemistry, University of Bristol, School of Medical Sciences, University Walk, Bristol, UK

**Keywords:** Lactate transport, MCT1, CD147, basigin, monocarboxylate transport, fluorescence resonance energy transfer, protein/protein interaction, site-directed mutagenesis, Xenopus oocytes

## Abstract

Monocarboxylate transporters MCT1-MCT4 require basigin (CD147) or embigin (gp70), ancillary proteins with a glutamate residue in their single transmembrane (TM) domain, for plasma membrane (PM) expression and activity. Here we use site-directed mutagenesis and expression in COS cells or *Xenopus* oocytes to investigate whether this glutamate (Glu_218_ in basigin) may charge-pair with a positively charged TM-residue of MCT1. Such residues were predicted using a new molecular model of MCT1 based upon the published structure of the *E. coli* glycerol-3-phosphate transporter. No evidence was obtained for Arg_306_ (TM 8) of MCT1 and Glu_218_ of basigin forming a charge-pair; indeed E218Q-basigin could replace WT-basigin, although E218R-basigin was inactive. No PM expression of R306E-MCT1 or D302R-MCT1 was observed but D302R/R306D-MCT1 reached the PM, as did R306K-MCT1. However, both were catalytically inactive suggesting that Arg_306_ and Asp_302_ form a charge-pair in either orientation, but their precise geometry is essential for catalytic activity. Mutation of Arg_86_ to Glu or Gln within TM3 of MCT1 had no effect on plasma membrane expression or activity of MCT1. However, unlike WT-MCT1, these mutants enabled expression of E218R-basigin at the plasma membrane of COS cells. We propose that TM3 of MCT1 lies alongside the TM of basigin with Arg_86_ adjacent to Glu_218_ of basigin. Only when both these residues are positively charged (E218R-basigin with WT-MCT1) is this interaction prevented; all other residue pairings at these positions may be accommodated by charge-pairing or stabilization of unionized residues through hydrogen bonding or local distortion of the helical structure.

## Introduction

The rapid proton-linked transport of monocarboxylates such as lactate and pyruvate across the plasma membrane plays a critical role in the metabolism and pH regulation of most cells (Poole & Halestrap 1993). Monocarboxylate transport is mediated by a family of monocarboxylate transporters (MCTs) of which there are now known to be 14 members, although only for members 1–4 has lactate and pyruvate transport been demonstrated directly (Halestrap & Price 1999, Halestrap & Meredith 2004). The MCTs are 12 transmembrane domain (TM) proteins with most sequence variation found in the long intracellular loop between TMs 6 and 7, the short N-terminal domain and the longer intra-cellular C-terminal domain (Halestrap & Price 1999). In order to be translocated correctly to the plasma membrane, MCTs 1–4 require the ancillary protein basigin (also known as CD147) or its homologue, embigin (also known as gp70) (Poole & Halestrap 1997, Kirk et al. 2000, Philp et al. 2003). These two proteins are closely related and comprise a single TM containing a conserved glutamate residue, a short intracellular C-terminal domain and a larger extracellular N-terminal domain containing 2 or 3 immunoglobulin folds (Muramatsu & Miyauchi 2003). Co-immunoprecipitation studies have shown that basigin, rather than embigin, normally associates with both MCT1 and MCT4 in the plasma membrane of most tissues. However, embigin can fulfil this role when no basigin is expressed, as is the case in erythrocytes of some species such as the rat, and is essential for MCT2 expression (Kirk et al. 2000; Wilson et al. 2005). Continued association between MCT1 or MCT4 and its ancillary protein is required for transport activity, and disruption of this interaction by covalent modification of basigin with the organomercurial reagent p-chloromercuribenzene sulphonate (pCMBS), inhibits transport (Wilson et al. 2005). We have also used fluorescence resonance energy transfer (FRET) between MCTs tagged with cyan-fluorescent protein (CFP) and basigin or embigin tagged with yellow-fluorescent protein (YFP) to confirm the continuing interaction of proteins within the plasma membrane (Wilson et al. 2002).

Using chimeras of CD2 and basigin we have shown that the extracellular domain of the ancillary protein is not involved in its interaction with MCTs (Kirk et al. 2000). Thus an interaction between the TM of basigin or embigin and TM (s) of the MCTs seems likely. This would be consistent with the presence of a glutamate in the TM of basigin, since this is unusual in a single-pass (monotopic) protein (Green 1991), and may reflect the formation of a charge-pair with a positive residue in a TM of the MCT. Our earlier sequence-based structural predictions (Halestrap & Price 1999) combined with the site-directed mutagenesis studies of Stefan Bröer's laboratory (Rahman et al. 1999, Galic et al. 2003) implicate Arg_306_ in TM 8 (numbering based on rat MCT1) as a potential candidate for such a charge-pair and this provided the initial focus for the studies reported here. However, structural predictions (Wilson et al. 2005) based around the published crystal structures of two members of the major facilitator superfamily, the *E. coli* glycerol-3-phosphate transporter (GlpT) and lactose permease (LacY) (Huang et al. 2003, Abramson et al. 2003) suggest that there may be other positively-charged residues in other TM domains of MCT1 capable of forming charge-pairs with the TM glutamate of basigin. These are Arg_86_, Arg_196_ and His_337_. Prior to the ability to model MCT1 upon the *E. coli* glycerol-3-phosphate transporter (GlpT) and lactose permease (LacY) Bröer's group had mutated potential TM charged resides to investigate their importance in MCT1 translocation and activity (Rahman et al. 1999, Galic et al. 2003). These studies showed that mutation of His_337_ to glutamine was without effect on MCT1 targeting to the plasma membrane and its activity (Rahman et al. 1999). They also showed that the same was true for Lys_137_, Lys_141_, Lys_142_, but that R143Q-MCT1, whilst correctly targeted, was inactive. These data suggest that the positive charge of Arg_143_ may be important for activity but not plasma membrane expression (Rahman et al. 1999, Galic et al. 2003). This conclusion was strengthened by the observation that the R143H mutant was active (Rahman et al. 1999). In this paper we investigate whether Arg_86_ and Arg_196_ may play a critical role in MCT1 activity or interaction with basigin and also provide a more rigorous structural model for MCT1 with which to interpret our data.

## Materials and methods

All reagents were obtained from Sigma (Poole, UK) unless otherwise stated. Polyclonal antibodies against the C-terminal 16 amino acids of rat MCT1 and human basigin were raised in rabbits as described previously (Poole et al. 1996, Wilson et al. 2005). The mouse mAb RET.PE-2 recognizing rat basigin (Finnemann et al. 1997) was a generous gift of Dr Neil Barclay (University of Oxford). Secondary antibodies for western blotting were from Amersham Biosciences (anti-rabbit) and Sigma (anti-chicken). An antisense nucleotide (TTCTCATAAATAAAGATTATTGTG) against *Xenopus laevis* basigin was chemically synthesized (Wilson et al. 2005).

### Cloning of cDNA constructs

The coding regions of human basigin and rat MCT1 were sub-cloned into the *EcoRI* site of the pCI-neo mammalian expression vector (Promega) and the *Xenopus* oocyte vector *pGHj* as described elsewhere (Manning Fox et al. 2000, Wilson et al. 2005). The pCI-neo constructs required to express rat MCT1 and human basigin tagged on the N- and C-termini with CFP or YFP were produced as described previously (Wilson et al. 2002, Wilson et al. 2005).

### Site-directed mutagenesis

Site-directed mutagenesis (SDM) of MCT1 and basigin within the relevant vector was performed using the QuikChange kit (Stratagene, UK) and the presence of the desired mutation confirmed by sequencing. Primers containing the desired mutation were designed to be between 25 and 45 bases in length with a melting point greater that 78°C and are shown in Supplementary online data Table I. Typically, for mutant MCT1, thermocycling was performed using the following parameters: 30 sec at 95°C, 30 sec at 55°C and a 4.8 min extension time at 68°C. PCR conditions for creating mutant basigin were similar but with an extension time of 4 min.

**Table I:** Summary of the expression and transport activity of wild-type and mutant MCT1 expressed in *Xenopus laevis* oocytes in the presence and absence of WT- and mutant basigin. Where given, V_max_ and K_m_ values (±SE) were determined from the initial rates of transport of L-lactate determined using BCECF fluorescence as described under Methods. Four oocytes were used in each case and L-lactate was added at 2.5, 5, 10, 20 and 40 mM. Data analysis was performed as described previously (Manning Fox et al. 2000). Where no transport could be detected using BCECF confirmation was made by determining the uptake of 0.5 mM L-[^14^C]-lactate at 10 min as described under Methods. Transport was indicated when uptake was significantly greater than in water injected oocytes, but in no case in which no BCECF response was detected was this observed. nd: not determined.

	Transport measured using BCECF			Plasma membrane expression determined using:		
		
Oocyte injection	Detectable	K_m_±SE (mM)	V_max_±SE (ΔF.min^−1^)	Western blot	Sections
WT-MCT1	Yes	3.52±0.23	0.42±0.007	Yes	Yes
R306E-MCT1	No	–	–	No	No
D302R/R306E-MCT1	No	–	–	Yes	Yes
R306K-MCT1	No	–	–	Yes	Yes
WT-MCT1+WT-basigin	Yes	2.94±0.16	0.36±0.005	Yes	Yes
WT-MCT1+E218Q-basigin	Yes	2.73±0.21	0.48±0.009	Yes	Yes
R306E-MCT1 + E218R-basigin	No	–	–	No	No
D302R/R306E-MCT1 + E218R-basigin	No	–	–	Yes	Yes
R306K-MCT1 + E218R-basigin	No	–	–	Yes	Yes
R86Q-MCT1	Yes	8.79±1.5	0.42±0.034	Yes	nd
R86E-MCT1	Yes	6.48±1.5	0.67±0.050	Yes	nd

### Expression of basigin and MCT1 in Xenopus oocytes

Preparation of oocytes from mature adult female *Xenopus laevis* was performed by a modified technique that was found to improve stability following microinjection. The ovarian lobes were removed, transferred to a Petri dish containing sterile-filtered zero Ca^2+^ ND96 solution (93.5 mM NaCl, 2 mM KCl, 2.96 mM MgCl_2_ and 5 mM HEPES pH 7.4) and cut into 0.5 cm^2^ pieces. Cut lobes (5 g) were then placed into a 50 ml plastic tube and washed for 6 × 15 min in 20 ml of zero calcium ND96 solution on a rotating wheel (approx. 5 revs/min) prior to digestion in 20 ml sterile-filtered zero calcium ND96 containing 8 mg/ml Type I collagenase for 30 min on a rotating wheel. The digested lobes were washed with 3 × 20 ml zero calcium ND96, and then subject to another 30 min collagenase digestion before washing with 4 × 40 ml zero calcium ND96. The resulting oocytes were placed into a fresh 50 ml tube containing 40 ml of normal calcium (1.8 mM) ND96 solution and washed a further four times. Finally, oocytes were transferred to a Petri dish containing sterile filtered OR3 medium (200 ml Leibovitz L-15 medium, 136.6 ml milli Q water and 3.4 ml 100 × penicillin/streptomycin) and incubated at 18°C overnight prior to injection. cRNA was prepared by *in vitro* transcription from the appropriate linearized pGHJ plasmid (mMessage mMachine, Ambion, Texas, USA). Oocytes were injected with 10–25 ng of the relevant cRNA (MCT, embigin or basigin) and, where required, 200 ng of antisense cDNA against *Xenopus* basigin (see Wilson et al. 2005). Controls received the equivalent volume (14 nl) of water. Oocytes were then cultured in OR3 medium for at least 48–72 hrs with fresh medium each day.

### Measurement of L-lactate transport into Xenopus oocytes

Measurements were either made by following the change in intracellular pH using the ratiometric pH-sensitive fluorescent dye 2′-7′-bis(carboxyethyl)-5-6-carboxy-fluorescein (BCECF) as described previously (Manning Fox et al. 2000, Wilson et al. 2005) or by determining the rate of uptake of L-[^14^C]-lactate. For the latter protocol, 4 6 oocytes were placed in 1.5ml microcentrifuge tubes containing 100 μl uptake buffer (75 mM NaCl, 2 mM KCl, 0.82 mM MgCl_2_, 1 mM CaCl_2_, 20 mM Tris/HEPES, pH 7.4) and allowed to equilibrate for 1 min. The uptake solution was then removed by aspiration and replaced with 100 μl uptake buffer containing L-[^14^C]-lactate (0.5 mM, 7.4 MBq/ml). Following incubation for the required time at room temperature (22–25°C), the oocytes were rapidly washed five times with ice-cold uptake buffer. After the final wash each egg was transferred into a scintillation vial and homogenized in 100 μl 2% (w/v) SDS by vigorous pipetting prior to addition of 10 ml of scintillation fluid (Emulsifier-Safe, Perkin Elmer) and assay of [^14^C] by scintillation counting.

### Detection of MCT1and basigin expression by western blotting and immunofluorescence microscopy

A crude oocyte membrane preparation was used for detection of MCT1 and basigin expression by SDS-PAGE and western blotting as described previously (Friesema et al. 2003). For immunofluorescence, each oocyte was embedded in a 1 cm^3^ block of chicken liver with O.C.T embedding compound and snap frozen using liquid N_2_ cooled pentane. These blocks were then frozen to chucks with CRYO-MBED embedding compound using cryospray at approximately −52°C, and sections were cut in a cryostat at a thickness of 5 μm using a steel blade. Sections were transferred onto multispot microscope slides and fixed by placing in a chamber containing ice-cold acetone for 15 mins. Immunofluorescence microscopy was performed using the appropriate antibodies as detailed elsewhere (Friesema et al. 2003).

### Expression of MCT1 and basigin constructs in COS-7 cells for immunofluorescence microscopy, live cell imaging and FRET

COS-7 cells were transfected with the required constructs using Fugene 6 and incubated in a CO_2_ incubator for 48 h prior to measurement of protein expression. This was performed by immunofluorescence in fixed cells (Wilson et al. 1998) or directly in live cells expressing CFP- and YFP-tagged proteins where fluorescence resonance energy transfer (FRET) was also determined using a Leica confocal imaging spectrophotometer system (TCSSP2) attached to a Leica DMIRBE inverted epifluorescence microscope (Wilson et al. 2002, Wilson et al. 2005). Quantification of FRET was performed by calculation of the ratio of the fluorescence intensity emission signal at 480 nm to that at 530 nm when excited at the CFP excitation wavelength (430 nm). In all cases a 63 × 1.32 Na oil immersion objective was employed. When required, crude plasma membrane fractions were prepared from cultured cells in the presence of protease inhibitors as described previously (Wilson et al. 2005).

### Modelling the structure of MCT1

**Figure 1:**
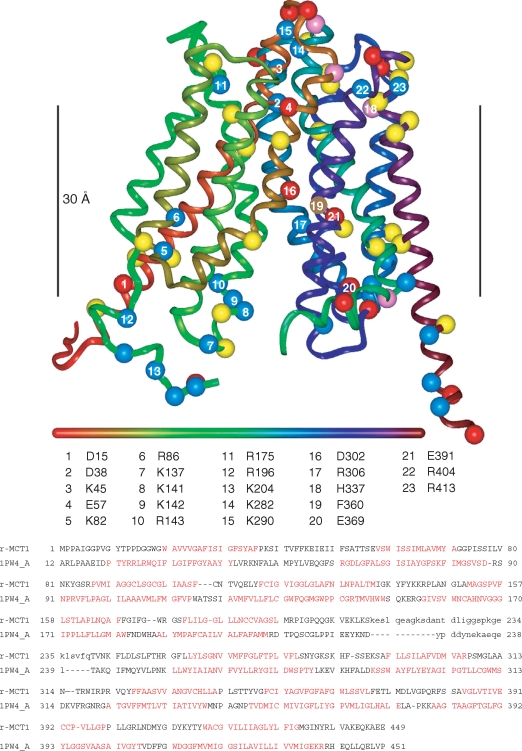
Homology model of rat MCT1 based on the *E. coli* glycerol 3-phosphate transporter (1PW4) template. The ribbon is coloured from red to purple along the sequence according to the horizontal bar (N to C). The numbered residues refer to those discussed in the text. Acidic residues (Asp, Glu) are represented by red spheres at the C-alpha position, basic residues (Arg, Lys) by blue spheres, histidine by pink spheres and aromatic residues frequently found at the phospholipid interface (Tyr, Trp) in yellow. Phe_306_, that is known to be in the substrate-binding pocket, is shown in brown. The black vertical bar measures 30 Å and marks the ‘best guess’ position of the lipid bilayer. The sequence alignment of rat MCT1 with the *E. coli* glycerol 3-phosphate transporter used to generate the model is shown beneath the model. Lower case letters refer to residues not built in the model and residues not present in the *1PW4* crystal structure respectively. Sequence in red refers to TM-helices in the template and predicted by TMHMM in the target sequence.

MCT sequences are only 10 15% identical to the two members of the superfamily whose crystal structures have been published, *E. Coli* glycerol-3-phosphate transporter (GlpT-1PW4, 3.3 Å; Huang et al. 2003) and lactose permease (LacY-1PV6, 3.5 Å; Abramson et al. 2003). Such low sequence identity (within the ‘random zone’) would typically preclude homology modelling. However, the MCT sequences contain an extra layer of information in the form of Trans-Membrane (TM) sequence signals that allow coarse registration of the target onto the template. These TM signals also afford sufficient amino-acid similarities (rather than identities) for alignment programs to find reasonably gap-free alignments of the MCT family with the template sequences. Initially, we used the alignments provided by Cn3D (http://www.ncbi.nlm.nih.gov/Structure/CN3D/cn3dtut.shtml) to produce a provisional model of the MCT1, MCT2 and MCT4 structures and encouragingly, the models built on the two templates superimposed reasonably well (Wilson et al. 2005). In this study we have refined this model by using other alignment software such as ClustalW, matching the TM sequence signals predicted by TMHMM to TM-helices in the templates, and finally by using molecular graphics (InsightII, Accelrys) to modify the registration of the target sequence on helices in the template structure to best accommodate residue sidechains. The modelling process yielded the final sequence alignment shown in Figure 1. All loop insertions and deletions were modelled apart from the large loop between the N-terminal and C-terminal domains of the protein. Any remaining sidechain clashes were relieved by appropriate rotamer choices made by inspection. Further refinement of the model was peformed by energy minimisation (Discover 2.95, Accelrys) while restraining the backbone to the corresponding template backbone. While analysis of the model showed it to possess acceptable stereochemical properties (Procheck) we still consider the model obtained here to be of ‘intermediate’ quality. Although considerable uncertainty remains over the precise registering of residues along the TM-helices, as might be expected from the low sequence identities, the model exhibits several features consistent with known experimental data from site-directed mutagenesis as will be described more fully in the Discussion. Thus it is likely to be a good predictor of the location of charged residues in TM regions.

## Results

### The glutamate in the TM of basigin is not critical for activity

**Figure 2:**
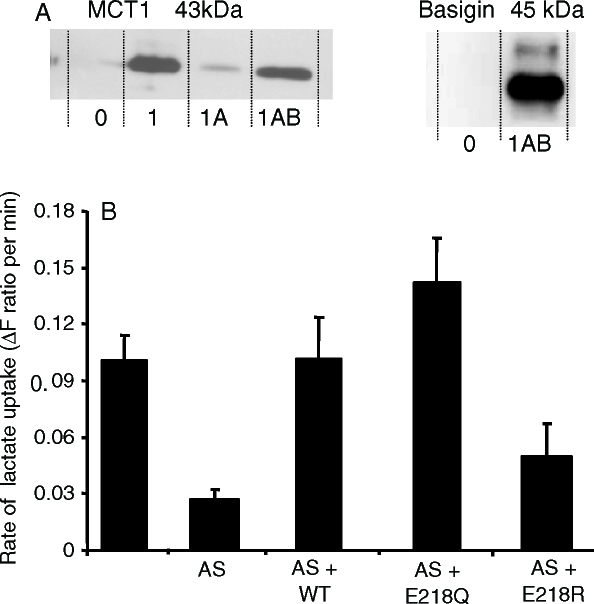
The E218Q mutant of basigin, but not the E218R mutant, supports lactate transport by MCT1 in Xenopus oocytes. In panel A, oocytes were injected with water (0), or cRNA for MCT1 (1) in the absence or presence (A) of antisense cRNA against *Xenopus* basigin and cRNA for rat basigin (B). Western blots are shown for the crude plasma membrane fraction using both MCT1 and basigin antibodies. In panel B rates of L-lactate (30 mM) transport into oocytes measured using BCECF fluorescence are shown as means±SEM of 5–8 separate oocytes. Where indicated, antisense (AS) against *Xenopus* basigin as well as the cRNA for WT-, E218Q- or E218R-basigin was co-injected with the MCT1 cRNA.

We have previously proposed that Glu_218_ in the TM of basigin might form a charge-pair with a positively charged residue in a TM of its partner MCT (Kirk et al. 2000, Halestrap & Meredith 2004). We first tested this possibility by mutating this glutamate to the uncharged glutamine or to the positively charged arginine and determining the ability of the mutants to support expression of active MCT1 in *Xenopus* oocytes. These cells do not express endogenous MCT activity, but they do possess an endogenous basigin that can support the expression of exogenous MCT1 from injected cRNA. This expression can be largely prevented by using antisense cRNA to knock down the endogenous basigin (Wilson et al. 2005). The western blots of Fig. 2A confirm that MCT1 expression was greatly reduced by the basigin antisense cRNA and that this was associated with a major reduction of lactate transport into the oocytes (Figure 2B). As predicted, when cRNA for rat WT-basigin was also injected, transport activity was restored (Figure 2B) and this was associated with MCT1 and basigin expression in the membrane fraction (Figure 2A). Transport activity was also restored when cRNA for the E218Q mutant of basigin was co-injected but E218R-basigin did not restore activity the small effect being statistically insignificant (Figure 2B). The large variation in rates of lactate transport seen when oocytes were injected with rat basigin cRNA together with antisense cRNA against *Xenopus* basigin (reflected in the large SEM), is thought to reflect incomplete endogenous basigin knockdown. Unfortunately we do not have a suitable antibody to confirm this.

**Figure 3:**
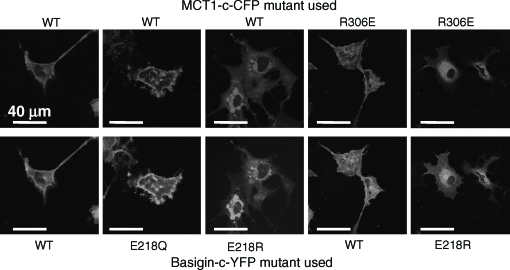
The E218Q mutant of basigin, but not the E218R mutant, is correctly targeted to the plasma membrane of COS cells. COS cells were co-transfected with MCT1-c-CFP and basigin-c-YFP constructs containing the mutations indicated and live cell imaging performed as described under ‘Methods’. This Figure is reproduced in colour in *Molecular Membrane Biology* online.

In Figure 3 we confirm these data in COS cells transfected with cDNAs encoding basigin tagged on the C-terminus with YFP (basigin-c-YFP) and MCT1 tagged on the C-terminus with CFP (MCT1-c-CFP). The presence of both proteins at the membrane was confirmed with live cell confocal imaging. Once again the E218Q basigin mutant was correctly targeted to the plasma membrane with MCT1 whilst the E218R mutant was largely retained in the perinuclear region. FRET confirmed that the E218Q-basigin-c-YFP was interacting with MCT1-c-CFP at the plasma membrane, the ratio of the fluorescence intensity emission signal at 480nm to that at 530nm when excited at the CFP excitation wavelength (430nm) being 0.833±0.047 (mean±SEM, *n* = 17) compared with a value of 0.7999±0.037 (*n* = 23) for the WT-basigin-c-YFP in the same experiment. By contrast, for the E218R-basigin-c-YFP co-expressed with MCT1-c-CFP the ratio was 1.034±0.032 (*n* = 12) that is similar to the value of 1.118±0.015 (*n* = 25) obtained using basigin-n-YFP co-expressed with MCT1-c-CFP. In the latter case, FRET is not possible because the YFP and CFP are on opposite sides of the plasma membrane and the ratio is similar to that for CFP expressed alone (Wilson et al. 2002, Wilson et al. 2005). Taken together, these data imply that the negative charge of basigin E_218_ is not critical for its ability to correctly chaperone MCT1 to the plasma membrane, although replacement with a positive charge is not tolerated.

### Arg_306_ is essential for MCT1 activity and forms a charge-pair with Asp_302_ in TM 8 of MCT1 but not Glu_218_ of basigin

**Figure 4:**
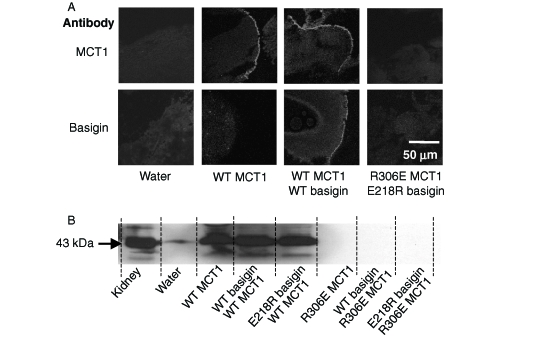
R306E-MCT1 and E218R-basigin are not expressed at the plasma membrane of Xenopus oocytes. Oocytes were micro-injected with the cRNA shown and after 72 hours some oocytes were used for immunofluorescence microscopy with the antibody shown (panel A) and others used for membrane preparation followed by SDS-PAGE (20 mg protein) and western blotting with anti-rat MCT1 antibody (panel B). For the western blot, kidney plasma membranes were used as a positive control. The faint band in the water-injected controls represents very slight sample contamination and is only visible because of the over-exposure of the blot to ensure any expressed MCT was detected. Further details are given under ‘Methods’. This Figure is reproduced in colour in *Molecular Membrane Biology* online.

The cRNA encoding both WT- and R306E-MCT1 was microinjected into *Xenopus* oocytes in the presence and absence of cRNA for either WT- or E218R-basigin. Western blotting of a membrane fraction (Figure 4B) and immunofluorescence microscopy of oocyte sections (Figure 4A) demonstrated that only WT-MCT1 and WT-basigin, but neither R306E-MCT1 nor E218R-basigin, was expressed at the plasma membrane of the oocytes. This was true whatever combination of proteins was expressed, demonstrating that a charge swap was not tolerated. The same result was obtained in COS cells transfected with the CFP- and YFP-tagged MCT1 and basigin mutants (Figure 3). In a separate series of experiments we confirmed these data using MCT1 tagged with CFP on the N-terminus rather than the C-terminus. Here FRET measurements were also performed and these confirmed the lack of interaction of R306E-MCT1-n-CFP with either WT- or E218R-basigin-c-YFP. Mean 480:530nm fluorescence emission ratio values (±SEM; *n* = 8) of 1.29±0.042 and 1.30±0.084 were obtained compared with 0.827±0.063 for the control FRET pair (WT-MCT1-n-CFP with WT-basigin-c-YFP) and 1.143±0.017 for the non-FRET pair (WTMCT1-n-CFP with WT-basigin-n-YFP).

Yet further confirmation for the inability of R306E-MCT1 to be properly expressed at the plasma membrane was obtained by measurement of L-lactate transport into *Xenopus* oocytes using BCECF fluorescence. Thus oocytes injected with cRNA for R306E-MCT1 gave no lactate induced pH change whether or not co-injected with cRNA for E218R-basigin (data not shown). Nor did the E218R-basigin inhibit expression of WT-MCT1 at the plasma membrane (Figure 4B), implying that it was not competing with endogenous basigin. This was also confirmed with transport measurements where V_max_ values (±SE for 4 eggs) determined in the absence and presence of E218R basigin were 0.42±0.007 and 0.48±0.009 fluorescence ratio units per min respectively, with corresponding K_m_ values of 3.52±0.23 and 2.73±0.21 mM. These data are summarized in Table I.

**Figure 5:**
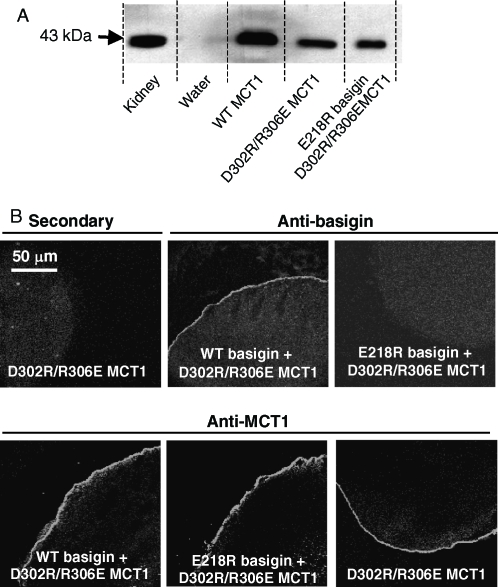
D302R/R306E-MCT1 is expressed at the plasma membrane of Xenopus oocytes but is inactive. Details are as given for Figure 4. Transport measurements are not shown because D302R/R306E-MCT1 failed to elicit any lactate transport whether or not WT- or E218R-basigin cRNA was co-injected. This Figure is reproduced in colour in *Molecular Membrane Biology* online.

It has been suggested that Arg_306_ of MCT1 is likely to form a charge-pair with Asp_302_ within TM 8 (Rahman et al. 1999). Although our data rule out a simple charge-pair between R_306_ of MCT1 and Glu_218_ of basigin, it remains a possibility that a more complex charge interaction occurs within the membrane that also involves Asp_302_. Thus we created the double mutant D302R/R306E-MCT1 and looked at its expression in *Xenopus* oocytes. In Figure 5 we demonstrate by western blotting (panel A) and immunofluorescence microscopy (panel B) that, unlike the single R306E mutant, the double mutant was translocated to the plasma membrane. As found for WT-MCT1 (Figure 4B), co-injection of either WT- or E218R-basigin cRNA was without effect on D302R/R306E MCT1 expression. However, we were unable to demonstrate any lactate transport catalysed by the expressed D302R/R306E MCT1, whether or not cRNA for WT- or E218R-basigin was co-injected. This was confirmed using both the radioactive and the BCECF fluorescence transport assays (Table I). These data suggest that a charge-pair between Asp_302_ and Arg_306_ is essential for correct folding of MCT1 and its translocation to the PM, and that reversing the orientation of these groups is tolerated for folding/translocation but not for catalytic activity.

### R306K-MCT1 is expressed at the plasma membrane, but is catalytically inactive

**Figure 6:**
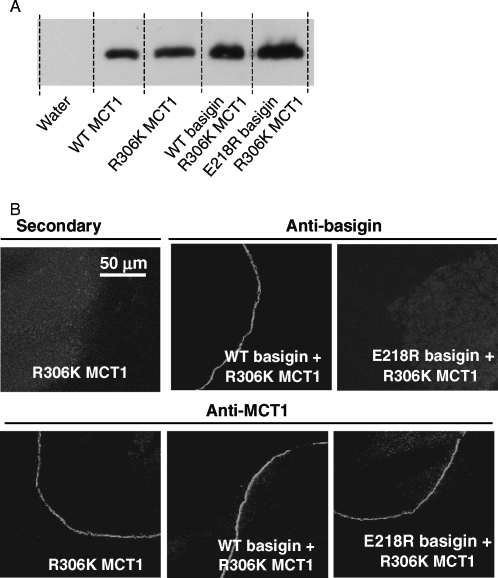
R306K-MCT1 is expressed at the plasma membrane of Xenopus oocytes but is inactive. Details are as given for Figure 4. Transport measurements are not shown because R306K-MCT1 failed to elicit any lactate transport whether or not WT- or E218R-basigin cRNA was co-injected. This Figure is reproduced in colour in *Molecular Membrane Biology* online.

The data presented above suggest that the precise geometry of Asp_302_ and Arg_306_ may be critical for the catalytic mechanism of MCT1, and this was further investigated by making the conservative change of Arg_306_ to a lysine. Western blotting and immunofluorescence microscopy confirmed that this mutant was translocated to the plasma membrane (Figure 6), yet once again we were unable to measure any lactate transport activity (data not shown). Taken together, these data (summarized in Table I) imply that not only is the correct orientation of Asp_302_ and Arg_306_ essential for the catalytic activity of MCT1 but that Arg_306_ plays a critical role that cannot be substituted by lysine.

### The role of other charged residues in transmembrane helices of MCT1

**Figure 7:**
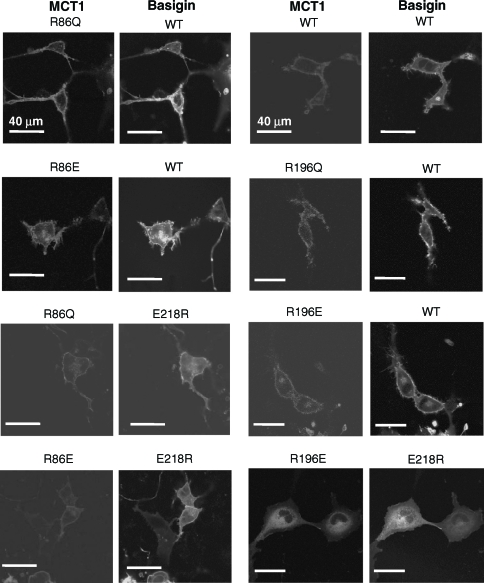
Mutation of Arg_86_ or Arg_196_ to glutamine or glutamate does not prevent MCT1 from being correctly targeted to the plasma membrane of COS cells. COS cells were co-transfected with MCT1-c-CFP and basigin-c-YFP constructs containing the mutations indicated and live cell imaging performed as described under ‘Methods’. This Figure is reproduced in colour in *Molecular Membrane Biology* online.

**Figure 8:**
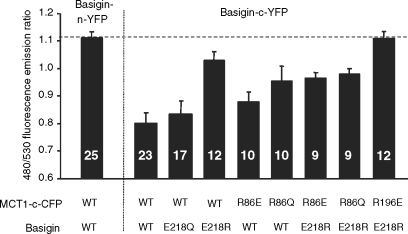
FRET measurements suggest that mutation of Arg_86_ perturbs the interaction of MCT1 with basigin. COS cells were cotransfected with MCT1-c-CFP and basigin-c-YFP constructs containing the mutations indicated and live cell imaging with determination of FRET performed as described under ‘Methods’. Data are presented as means±SEM for the number of observations shown.

Our structural predictions (Wilson et al. 2005) based around the published crystal structures of two members of the major facilitator superfamily, the *E. coli* glycerol-3-phosphate transporter (GlpT) and lactose permease (LacY) (Huang et al. 2003, Abramson et al. 2003) suggested that Arg_86_, Arg_196_ and His_337_ might also be candidate residues for forming a charge-pair with Glu_218_ in basigin. Of these, Bröer's group have already mutated His_337_ to glutamine and shown it to have no effect on either MCT1 targeting to the plasma membrane or its activity (Rahman et al. 1999). They also showed that the same was true for Lys_137_, Lys_141_, Lys_142_ and Arg_143_, although R143Q-MCT1 was inactive despite being correctly targeted, (Rahman et al. 1999, Galic et al. 2003). We have therefore focussed our attention on Arg_86_ and Arg_196_, mutating both of them to either glutamine glutamate. The R86Q-, R86E-, R196Q- and R196E-mutants of MCT1 were all active when expressed in *Xenopus* oocytes, and for R86Q-MCT1 and R86E-MCT1 the K_m_ values for L-lactate were determined and were not significantly different from WT-MCT1 (Table I). These data imply correct plasma membrane expression of the mutants in *Xenopus* oocytes. The same was true in COS cells as shown in Figure 7 where we used live cell imaging to demonstrate plasma membrane expression of MCT1-c-CFP when co-expressed with basigin-c-YFP. In these experiments we also co-expressed R86E-MCT1-c-CFP and R196E-MCT1-c-CFP with E218R-basigin. In the case of R196E-MCT1 no plasma membrane expression was observed as found previously for WTMCT1 (Figure 3). However, when either R86QMCT1-c-CFP or R86E-MCT1-c-CFP was co-expressed with E218R-basigin-c-YFP both proteins were expressed at the plasma membrane (Figure 7). The interaction between R86Q-MCT1 and R86E-MCT1 with E218R-basigin-c-YFP was studied further using FRET and the data are presented in Figure 8. Although not giving as much FRET as WT-MCT1-c-CFP with WT-basigin-c-YFP, the 480/530 fluorescence emission ratios for R86QMCT1-c-CFP (0.98±0.020) and R86E-MCT1-c-CFP (0.97±0.020) were significantly lower than that observed for the non-FRET pair MCT1-c-CFP with WT-basigin-n-YFP (1.18±0.015) in the same experiment. For R196E-MCT1-c-CFP expressed with E218R-basigin-c-YFP, there was no plasma membrane expression, and as predicted there was no FRET observed with the fluorescence emission ratio being 1.11±0.023.

For completion, we also mutated Lys_38_, Lys_45_ and Arg_404_ to glutamine or glutamate, but found that all of these mutants of MCT1 were expressed at the plasma membrane and showed catalytic activity. The results are summarized in Supplementary data Table II.

## Discussion

The data we present in this paper do not support our earlier proposal that the transmembrane glutamate (Glu_218_) of basigin must form a charge-pair with a positively charged residue in a TM of MCT1 since substituting this glutamate with the uncharged glutamine does not prevent basigin fulfilling its role. However, our data are consistent with an interaction between Glu_218_ in basigin and Arg_86_ in TM3 of MCT1 as will be discussed further below. Our data also confirm the importance of a charge-pair between Asp_302_ and Arg_306_ in TM 8 of MCT1.

### The validity of the model used to predict TM charges in MCT1

There remains considerable uncertainty over the precise registering of residues along the TM-helices in our revised model of the MCT1 structure (Figure 1), as might be expected from the low sequence identities between MCTs and the transporter used as the structural template (*E. coli* glycerol-3-phosphate transporter). Nevertheless, our proposed structure exhibits several features consistent with site-directed mutagenesis studies performed here and elsewhere. First, Arg_306_ and Asp_302_ in TM8 are properly aligned to form a charged pair as suggested by the observation that neither R306E nor D302R were expressed at the PM whereas the double mutant was (Figures 2 & 3 and Rahman et al. 1999). Second, F360C-MCT1 can transport bulkier monocarboxylates than WTMCT1 (Kim-Garcia et al. 1994), suggesting that Phe_360_ is in the substrate binding pocket. This residue is close to Arg_306_ and Asp_302_ in our model. Third, Galic et al. (2003) have identified another likely charge-pair, between Arg_143_ (loop between TM4 and TM5) and Glu_369_ (loop between TM10-TM11), where again reversal of the charges allowed expression but not activity. Their data suggested that this charge-pair breaks during the translocation cycle with a shift of TMs 8 and 10 and proton transfer from Asp_302_ to Glu_369_. Flexibility of helix 5 is required for this and our model allows it, although in the conformation shown, Glu_369_ is well separated from Arg_143_. However, it is at an appropriate position in the TM domain to allow such an interaction upon helix movement during the translocation cycle. We have previously shown a slight increase in FRET between MCT1-c-CFP and basigin-c-YFP upon substrate or inhibitor binding that is consistent with such a conformational change (Wilson et al. 2005). A feature of our new model is that Arg_196_, that we originally placed in TM 6, is now placed on the cytosolic surface of MCT1. This is consistent with the data we present here that show this residue to play no critical role in the interaction ofMCT1 with basigin or its catalytic activity.

### Glu_218_ in the transmembrane domain of basigin is not essential for its function

As noted in the Introduction, the presence of a charged residue such as glutamate in the transmembrane domain of a single pass protein is unusual (Green 1991) and thus might be expected to play a critical role in the function of basigin and embigin. However, our data show that it can be mutated to the uncharged glutamine and still interact with MCT1 at the plasma in Xenopus oocytes (Figure 2) and COS cells (Figure 3). In the former case, it was also possible to confirm that E218Q-basigin supported MCT1 catalytic activity (Figure 2). However, when Glu_218_ was mutated to the positively charged arginine, the protein was not properly targeted to the plasma membrane in either COS cells (Figure 3) or Xenopus oocytes (Figure 4). It was previously postulated that Glu_218_ of basigin might charge-pair with a positively charged residue in a transmembrane helix of MCT1, for which the obvious candidate is Arg_306_ (Wilson et al. 2002). This now seems unlikely since E218R-basigin could not be rescued by co-expressing with R306E MCT1 (Figures 3 and 4), yet it would be predicted that any charge-pair would be retained under these conditions, although with reverse polarity.

Two other strong candidates for positively charged residues in TM domains of MCT1 that might interact with Glu_218_ of basigin, identified from our structural model of MCT1 (Figure 1), are Arg_86_ and His_337_. However, Rahman et al. (1999) have shown that H337Q-MCT1 is expressed in active form at the plasma membrane ruling out such a role for this residue. Our own data (Figure 7 and Supplementary data Table II) allow us to draw the same conclusion for Arg_86_, since swapping the charge from positive to neutral (glutamine) or negative (glutamate) did not prevent the expression of active MCT1 at the plasma membrane. Overall, our data appear to rule out an essential charge-pair between Glu_218_ of basigin and a positively charged TM residue of MCT1 and would be compatible with Glu_218_ being protonated and thus uncharged within the membrane.

### Confirmation that Arg_306_ forms a charge-pair with Asp_302_ in TM 8 of MCT1

The proposal that Arg_306_ in TM 8 of MCT1 forms a charge-pair with Asp_302_ within the same TM as proposed by (Rahman et al. 1999) is supported by our model and here we have confirmed it experimentally. Thus when either residue was mutated on its own, the protein was poorly expressed at the plasma membrane (Figures 2–4), whereas the double mutant, with the charges swapped, did reach the membrane (Figure 5). However, the double mutant was found to be catalytically inactive, implying that the geometry of the interaction may be critical for correct function. Interestingly, Galic et al. (2003) have shown that Arg_143_ and Glu_369_ are also likely to form a charge-pair and here too, reversal of the charges allows correct folding and membrane translocation but not catalytic activity. Further evidence for the critical geometry surrounding the charge-pair of Asp_302_ with Arg_306_ if MCT1 is to be catalytically active is the observation of Rahman et al. (1999) that Asp_302_ cannot be replaced by the conservative change to a glutamate. Furthermore, our own data reveal that although the conservative replacement of Arg_306_ with lysine allows for expression at the plasma membrane (Figure 6), the protein is catalytically inactive (Supplementary data Table II). This is a similar situation to that found in lactate dehydrogenase where arginine is critical for substrate binding (Hart et al. 1987) and like lactate dehydrogenase, MCT1 is inhibited by the arginine-specific agent, phenylglyoxal (Donovan & Jennings 1986, Poole & Halestrap 1993).

### Where does basigin bind to MCT1?

Although our data argue against an essential interaction of Glu_218_ of basigin with a charged residue in a TM of MCT1, we do provide some evidence that Arg_86_ in TM3 of MCT1 may be in close proximity to Glu_218_ of basigin. This is implied by the data of Figure 7 showing that E218R-basigin can associate with R86E-MCT1 and R86Q-MCT1 at the plasma membrane, but not with WT-MCT1. This can be explained if TM3 of MCT1 lies alongside the TM of basigin, which would fit well with our structural model for MCT1 that places TM3 on the lipid-facing surface of MCT1. Arg_86_ is in the middle of TM3 where it could readily form a charge pair within the membrane with Glu_218_ in the middle of the TM of basigin. The same would be the case when the charges are swapped in cells expressing E218R-basigin with R86E-MCT1. However, when WT-MCT1 (with Arg at position 86) is expressed with E218R-basigin, association of the two proteins will be prevented by the presence of the two positive charges in close proximity in the membrane. Other combinations of the two residues that do not allow an appropriate charge-pair are tolerated as shown by co-expression of the proteins at the plasma membrane and their FRET, albeit less well than for the WT proteins (Figure 8). Under these circumstances the presence of an unpaired arginine or glutamate residue in the membrane may be enabled either by stabilization of the unionized residues through hydrogen bonding or through local distortion of the helical structure placing the tip of the charged residue outside of the hydrophobic region of the bilayer.

Additional evidence for the TM of basigin interacting with TM3 of the MCT comes from our earlier studies in which we demonstrated that substitution of cysteine with alanine or serine residues in TMs 3 and 6 of MCT4 increased its sensitivity to inhibition by p-chloromercuribenzene sulphonate (Wilson et al. 2005). This organomercurial reagent also prevented co-immunoprecipitation of MCT4 and MCT1 with basigin, consistent with the substitution of theses cysteine residues perturbing the interaction between basigin and the MCT. Our model suggests that TM6 lies adjacent to TM3 on the lipid-facing surface of MCT1 and thus it is possible that the TM helix of basigin lies between TMs 3 and 6 of MCT1. Further studies will be required to confirm these proposals.

## Abbreviations

BCECF: 2′-7′-bis(carboxyethyl)-5-6-carboxy-fluorescein
basigin-c-YFP: basigin tagged on the C-terminus with YFP
basigin-n-YFP: basigin tagged on the N-terminus with YFP
CFP: cyan variant of green fluorescent protein
MCT: monocarboxylate transporter
MCT1-c-CFP: MCT1 tagged on the C-terminus with CFP
SDM: site-directed mutagenesis
TM: transmembrane domain
WT: wild-type
YFP: yellow variant of green fluorescent protein.
